# The Application of Cation Exchange Membranes in Electrochemical Systems for Ammonia Recovery from Wastewater

**DOI:** 10.3390/membranes11070494

**Published:** 2021-06-30

**Authors:** Kai Yang, Mohan Qin

**Affiliations:** Department of Civil and Environmental Engineering, University of Wisconsin—Madison, Engineering Hall, 1415 Engineering Drive, Madison, WI 53706, USA; kyang288@wisc.edu

**Keywords:** cation exchange membrane (CEM), electrochemical systems, ammonia recovery, nitrogen recovery, wastewater treatment, ion transport

## Abstract

Electrochemical processes are considered promising technologies for ammonia recovery from wastewater. In electrochemical processes, cation exchange membrane (CEM), which is applied to separate compartments, plays a crucial role in the separation of ammonium nitrogen from wastewater. Here we provide a comprehensive review on the application of CEM in electrochemical systems for ammonia recovery from wastewater. Four kinds of electrochemical systems, including bioelectrochemical systems, electrochemical stripping, membrane electrosorption, and electrodialysis, are introduced. Then we discuss the role CEM plays in these processes for ammonia recovery from wastewater. In addition, we highlight the key performance metrics related to ammonia recovery and properties of CEM membrane. The limitations and key challenges of using CEM for ammonia recovery are also identified and discussed.

## 1. Introduction

Ammonia (NH_3_) is essential for producing nitrogen fertilizers and is also one of the most common industrial chemicals [1]. Ammonia is synthesized using the energy-intensive Haber-Bosch process [2,3], accounting for more than 1% of global energy consumption [4]. However, 30% of ammonia from the Haber-Bosch process is not utilized and ends up in wastewater [5]. Wastewater treatment plants remove ammonium nitrogen by converting it back to nitrogen gas using nitrification-denitrification or anammox process [6], which requires energy consumption and potentially contributes to greenhouse gas emissions. Hence, directly recovering ammonia from wastewater is considered as a promising strategy to save energy from both the Haber-Bosch process for ammonia synthesis and ammonia removal in wastewater treatment plants, as well as produce nitrogen fertilizer for agricultural applications.

Recently, various electrochemical approaches, including bioelectrochemical system (BES), membrane electrosorption (MES), electrochemical stripping (ECS), and electrodialysis (ED), have been used to recover ammonium nitrogen from various types of wastewater (e.g., urine, livestock wastewater, and synthesized wastewater) [7,8]. In the electrochemical systems, electrons are transferred from the anode electrode to the cathode electrode across an external circuit, with either redox reactions or electrosorption occurring at the electrodes. The electrical field drives the ions in solution to transport towards the oppositely charged electrode to maintain electroneutrality [9]. When a cation exchange membrane (CEM) is placed between the anode compartment and cathode compartment, transport of ammonium ions and other cations across the membrane is achieved while the passage of anions is blocked. The transported ammonium ions can then be concentrated and recovered to produce fertilizers. In addition to ammonia recovery, electrochemical processes have also been considered as a promising strategy to recover other prime targets, including battery elements, metals, and organic compounds, which have been detailed in previous research [10,11,12,13].

CEMs are widely used to separate electrolyte solutions in electrochemical systems. CEM is a dense polymer layer that consists of crosslinked polymer chains fixed with negatively charged groups [14]. Ideally, the fixed functional groups inhibit the transport of anions, while transport of cations, such as ammonium ions and protons, are allowed [15]. Due to the relatively small hydrated ionic size (0.331 nm) and fast diffusivity in CEM, ammonium ions can be easily transported across the CEM and serve as excellent charge carriers in electrochemical systems [16,17]. Hence, the CEM plays a vital role in ammonia recovery from wastewater.

This paper provides a comprehensive review on the application of CEM in electrochemical systems for ammonia recovery from wastewater. Specifically, we introduce four major types of electrochemical processes, including bioelectrochemical systems, electrochemical stripping, membrane electrosorption, and electrodialysis. First, we elucidate the basic principles of these technologies and the contribution of CEMs in achieving ammonia recovery from wastewater. Next, we discuss the mathematical models and equations addressing the ion transport phenomena in the electrochemical processes. We further explain the key parameters characterizing the systems. Then the corresponding CEMs and their properties are summarized and analyzed. The limitations and future perspectives of using CEM for ammonia recovery are identified and discussed.

## 2. Electrochemical Systems for Ammonia Recovery

### 2.1. Bioelectrochemical System (BES)

**Figure 1:**
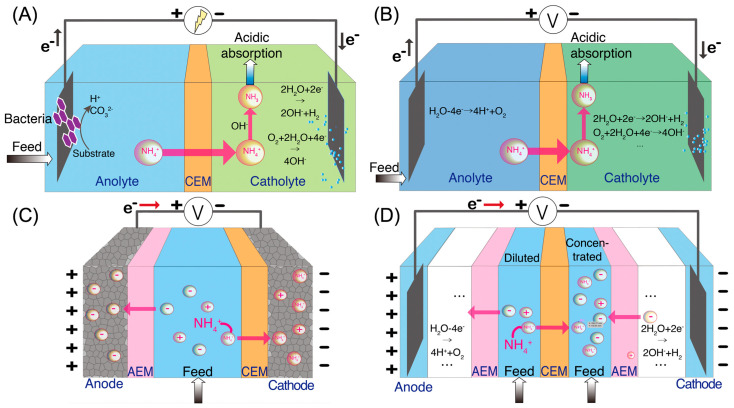
Schematic of four types of ammonia recovery technologies: (**A**) bioelectrocehmical system (BES), (**B**) electrochemical stripping (ECS), (**C**) membrane electrosorption (MES), and (**D**) electrodialysis (ED).

Bioelectrochemical system (BES) is a group of technologies that rely on the electrochemical interaction of microorganisms and electrodes (Figure 1A) [18,19,20,21]. In BES, organic compounds in wastewater are oxidized by exoelectrogens growing on the anode electrode, producing electrons that can be transferred to the cathode electrode through an external circuit [22]. When a cation exchange membrane (CEM) is placed between the anode compartment and cathode compartment, ammonium ions in the wastewater serve as charge carriers in the aqueous phase to maintain the charge balance [23]. In the cathode side, the ammonium ions are converted to ammonia gas due to the high pH value of the catholyte. The high pH, which has been shown to be sufficient for the conversion of ammonium ions to ammonia, is the result of hydroxide formation from reduction reactions, such as hydrogen evolution reaction (HER) and oxygen reduction reaction (ORR) [24]. The ammonia gas can then be recovered as fertilizers using various kinds of approaches (e.g., direct collection, acid absorption, and gas membrane collection) [25,26,27,28]. Ammonia recovery has been achieved in several kinds of BES, including microbial fuel cells (MFCs) and microbial electrolysis cells (MECs) [20,29]. In addition to ammonia recovery, energy can also be recovered in the form of electricity in MFCs autonomously and as hydrogen gas in MECs with an external power supply [30].

### 2.2. Electrochemical Stripping (ECS)

Electrochemical stripping (ECS) is another effective method to extract ammonium nitrogen from wastewater [8,26]. Similar to BES, ECS has an anode compartment and a cathode compartment, separated by CEM (Figure 1B). ECS requires an external power source to drive the electrochemical reactions. In ECS, the anode reaction can be an oxygen evolution reaction or hydrogen oxidation reaction, while the cathode reaction is either ORR or HER [8]. Since there is no microbial activity involved, the current density in ECS is not regulated by the microbial metabolism and community structure, allowing for a higher current density and a higher ammonia recovery rate than in BES [31]. However, compared to BES, higher energy consumption in ECS is expected.

### 2.3. Membrane Electrosorption (MES)

Membrane electrosorption (MES), which is also known as membrane capacitive deionization (MCDI), can selectively recover ammonium nitrogen from wastewater [32,33,34,35,36]. In the MES, a small voltage (<1.5 V) is applied between two porous carbon or battery electrodes (Figure 1C). The positively charged electrode and negatively charged electrode are equipped with an anion exchange membrane (AEM) and a CEM, respectively, to prevent the adsorption of co-ions and therefore improve the charge efficiency of MES [37,38,39]. The electric field generated from the applied potential drives the ions towards the oppositely charged electrodes, where they are immobilized in the micropores of the electrodes [40]. The key feature of MES is the capability of storing the ions in either electrical double layers (EDLs) of the electrode micropores or lattice structures. When wastewater is used as the feed solution, ammonium ions are separated from the feed solution and held at the EDLs or lattice structures of the electrode until the applied voltage is reversed or removed. Once the applied voltage is removed or reversed, the ions are released from the EDLs back into the solution, producing a concentrated stream and regenerating the electrode adsorption sites [41]. By sequentially extracting ammonium from the feed solution and draining ammonium to the concentrated stream, ammonia recovery is achieved.

### 2.4. Electrodialysis (ED)

**Figure 2:**
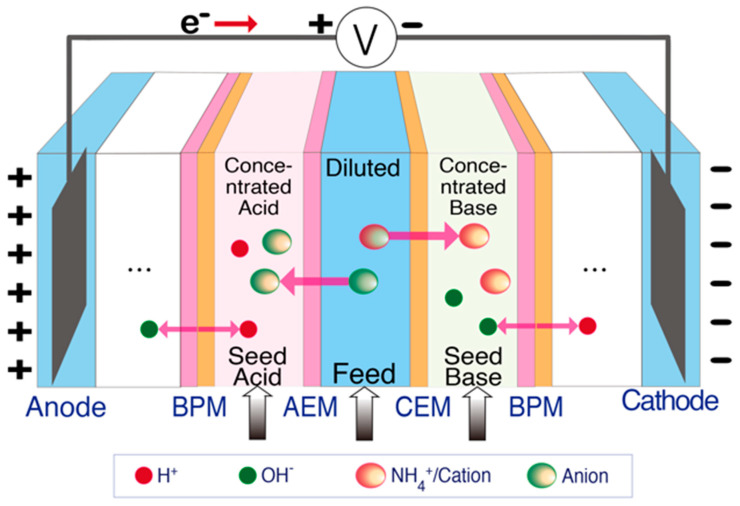
Schematic of BPM integrated ED for ammonia recovery.

Same as BES and ECS, electrodialysis (ED) relies on electrochemical redox reactions to generate an electric field and transport ions in solution through ion exchange membranes (IEMs) [42,43,44]. The main difference is that ED incorporates multiple pairs of ion exchange membranes between the anode and cathode electrodes (Figure 1D). When an electric field is applied, redox reactions occurring at the electrodes drive ions towards the oppositely charged electrodes. As ammonium-rich wastewater is passed through the channels between the alternating CEMs and AEMs, ammonium ions and anions are transported across the CEMs and AEMs, respectively, thereby generating diluted (ammonium removed) and concentrated (ammonium-rich) streams [15,44,45,46]. In addition, bipolar membranes (BPM) have been used together with IEMs in ED to enhance the reactor performance (Figure 2). A BPM is an ion exchange membrane that consists of an AEM layer and a CEM layer. It dissociates water molecules into protons and hydroxide ions in the presence of a strong electrical field [47,48,49,50]. The production of concentrated acid and alkali solution by BPM integrated ED facilitates ammonia recovery by the generation of NH_3_•H_2_O when treating wastewater [51,52].

## 3. Theory of Ion Transport

Mathematical models have been developed to describe and quantify the transport phenomena in the aforementioned processes [16,53,54,55], allowing for the prediction of ion concentration profiles and other key parameters that are difficult to measure using experimental study alone (e.g., potential inside the membrane) over a wide range of operating conditions. The mathematical models take into account the ion transport in both aqueous solutions and membranes, membrane properties, and redox reactions on the electrodes. Mass balance and electroneutrality are also included in the model. Usually, water transport through the CEM is neglected as the CEM is relatively thick and thereby has a high hydraulic resistance.

The Nernst Planck equation is widely used to describe ion flux, which is a combination of diffusion and electromigration, in both aqueous solutions and membranes [54,56,57]. The Nernst-Planck equation describes the flux of a specific ion as a function of concentration and potential gradient, which is calculated as:

(1) $$J_{i}=-D_{i}\cdot \frac{\partial c_{i}}{\partial x}-D_{i}\cdot z_{i}\cdot c_{i}\cdot \frac{\partial \phi }{\partial x}$$

where $J_{i}$ is the flux of ion *i*, $D_{i}$ is the diffusion coefficient of species *i*, $z_{i}$ is the ionic charge number, *c_i_* is the concentration, $\frac{\partial \phi }{\partial x}$ is the electrical potential gradient, and *x* is the vertical position from the electrodes.

The models consider mass conservation for all species. For a defined control volume, the mass variation for a specific species is equal to the sum of ion transport flux and the accumulation rate due to reactions. For instance, in the catholyte, the mass balance of species *i* is determined by:

(2) $$V\cdot \frac{dc_{i}}{dt}=A\cdot J_{i}+\sum_{j}v_{j}\cdot r_{j}\cdot V$$

where V is the volume of the catholyte, $\frac{dc_{i}}{dt}$ is the concentration variation with time, *A* is the surface area of control volume, *J_i_* is the ion transport flux of species *i*, $\sum_{j}v_{j}\cdot r_{j}\cdot V$ is the sum of generation or consumption rate of *i* from each reaction *j*, *v_j_* and *r_j_* represents the stoichiometric coefficient and reaction rate of each reaction involving *i*, respectively.

The ion concentration distribution at the membrane-solution interface is governed by Donnan equilibrium. The Donnan equilibrium describes an electrical potential drop at the membrane-solution interface as a function of the concentration of species *i* at the membrane side and solution side of the interface [58], which is calculated as:

(3) $$\Delta \phi =-\frac{RT}{z_{i}F}\cdot ln\frac{c_{i}^{m}}{c_{i}^{s}}\;$$

where R is the ideal gas constant, $c_{i}^{m}$ and $c_{i}^{s}$ is the concentration of ion *i* at the membrane side and at the solution side, respectively.

The charge transfer is always crucial in electrochemical systems. In the mathematical models, electrons flow from anode electrode to cathode electrode across external wires, while ions transport in the aqueous solution between the electrodes. The total charge transferred by ions has to be identical to the charge transferred by electrons, which is calculated as:

(4) $$I_{ext}=A_{m}\cdot F\cdot \sum_{i}J_{i}\cdot z_{i}$$

where $I_{ext}$ represents external current, *A_m_* is the membrane area, F is the Faraday constant, $\sum_{i}J_{i}\cdot z_{i}$ is the sum of charge flux for each species of ion *i*.

Additionally, the individual ion transport behavior is coupled by the law of electroneutrality, which is given by:

(5) $$\sum z_{i}\cdot c_{i}=0$$

(6) $$\sum z_{i}\cdot c_{i}+\omega \cdot X=0$$

where *ω* and *X* represent the charge of the ion exchange membrane and fixed ion concentration in the membrane, respectively. Here *ω* = −1 is applied for CEM since the charge of fixed groups in CEM is negative.

In BES and ECS, mathematical models based on the abovementioned equations have been applied to understand ammonium transport towards its recovery [16,55]. Previous modeling work has shown that the transport of ammonium ions accounts for approximately 90% of the total current in a MEC reactor fed with synthetic digestion effluent of livestock waste, indicating that ammonium ions can serve as proton shuttles for the charge transport across the CEM [16]. While the redox reactions acidify and basify anolyte and catholyte, respectively, the transport of ammonia and ammonium ions can buffer the pH in both anolyte and catholyte, leading to relatively stable pH values in both sides. The mathematical model was also applied to a MEC reactor to recover ammonia gas from synthetic urine [55]. The simulation results indicate that high current density is beneficial for ammonium transport across the CEM (as shown in Equation (6)), while charge density of CEM membrane has little impact on ammonium transport. When charge density of CEM membrane varies between 0 M and 4 M, the transport number of ammonium ions is always close to one, resulting in a large amount of ammonium flux from the anode chamber to the cathode, whereas the transport number of protons is close to zero. One major concern in BES is the back diffusion of ammonia ions from the cathode side to the anode side, which decreases the efficiency of ammonia stripping in the cathode. In other electrochemical systems (i.e., ED and MES), the ammonium transport has not been detailed analyzed using mathematical models, although mathematical models have been developed for these processes to capture the ion transport phenomena or estimate the energy consumption [53,54,59,60].

## 4. Key Performance Metrics and Membrane Properties

In electrochemical systems, pH plays a key role in ammonia recovery. Owing to the redox reactions happening at anode and cathode electrodes, the pH value in the anode chamber tends to decrease while the pH in the cathode chamber tends to increase when there are only limited pH buffer capacities in both sides. The pH value can not only affect the electrode potential, but also impact the partitioning of ammonium nitrogen between its two forms (i.e., ammonium and ammonia). In BES and ECS fed with ammonia-rich wastewater in the anode chamber, ammonium nitrogen is the major form in the anolyte. In the catholyte, the ammonium nitrogen usually exists as ammonia when the pH is higher than 10 since the pKa value of ammonia is 9.25 at 25 °C. As a result, the inherent pH gradient could create a concentration gradient for ammonium ions, which favors the transport of ammonium ions across the CEM membrane.

Among all the key performance metrics, transport number, a dimensionless parameter, is of great importance in electrochemical reactors. Transport number is used to directly describe the amount of ions that move across the membrane. The transport number of an ion species *i* is defined as the proportion of the charge carried by this kind of ions transported in the electrolyte to the total charge carried by all the ions transported between the electrolytes, which is given by [7,61,62]:

(7) $$t_{i}=\frac{z_{i}J_{i}}{\sum z_{i}J_{i}}=V\cdot F\cdot z_{i}\cdot \frac{c_{i}\left(0\right)-c_{i}\left(t\right)}{\int_{0}^{t}I_{tot}\;dt}$$

where $t_{i}$ represents the transport number of *i*, *J_i_* is the flux of the ion *i*, *z_i_* is the charge carried by ion *i*, *I_tot_* is the total current in the external circuit, $\int_{0}^{t}I_{tot}\;dt$ is the sum of charge transferred by the electrons at the external circuit within the time period of *t*, *V* is the volume of the electrolyte, *F* is the Faraday constant, *c_i_*(0) and *c_i_*(*t*) are the concentrations at time of zero and time of *t*, respectively. Owing to electroneutrality, the total charge carried by all the ions transported between the electrolytes is supposed to be equal to the external electrical current. In an electrochemical reactor, a larger transport number of ammonium nitrogen indicates more ammonium ions are transported across the CEM per unit current. Ideally, the transport of ammonium ions across the CEM dominates the ion transport across the membrane with a transport number close to one. A higher transport number of ammonium nitrogen benefits the system since it implies that more current is used to transport ammonium ions.

In a BES reactor fed with ammonia-rich livestock wastewater, the transport number of ammonium ions is reported up to 0.49 [7]. In a recent study, the transport efficiency of total ammonia nitrogen (TAN), which is the same as the transport number of TAN, was reported as 92% in a scaled-up BES system [63]. When calculating the TAN transport efficiency, the transport of ammonium ions and ammonia are lumped, regardless of the partition of ammonia and ammonium ions. The TAN transport number is reported to be affected by the load ratio, which is defined as the ratio of the applied current to the TAN loading rate [64]. By comparing the TAN transport number and the load ratio, the proportion of the current that is used for TAN transport can be calculated, and thus the transport of other cations, such as Na^+^ and K^+^, can also be evaluated.

Another performance metric is the selectivity of NH_4_^+^ over another ion M^n+^ existing in the solution, which is defined as [53,65]:

(8) $$\rho \left(\frac{NH_{4}^{+}}{M^{n+}}\right)=\frac{\Delta c_{NH_{4}^{+}}/c_{NH_{4}^{+},0}}{\Delta c_{M^{n+}}/c_{M^{n+},0}}$$

where $\Delta c_{NH_{4}^{+}}$ and $\Delta c_{M^{n+}}$ are the decreases in NH_4_^+^ and M^n+^ concentrations in the anode chamber, respectively, $c_{NH_{4}^{+},0}$ is the initial concentration of NH_4_^+^, and $c_{M^{n+},0}$ is the initial concentration of M^n+^ in the anode chamber. Selectivity is evaluated when comparing ammonium transport to the transport of other cations in the wastewater (e.g., Na^+^ and K^+^). For a fixed current, other cations in the anolyte can potentially compete with the ammonium ions for transport across the CEM membrane. A higher selectivity of ammonium ions to other cations implies more portions of ammonium nitrogen are transported across the CEM membrane, therefore potentially improving the removal of ammonium ions from the anolyte (i.e., wastewater). There is only limited discussion on the selectivity of ammonium ions across the CEM membrane in the references. More efforts are needed to investigate and understand ammonium selectivity in electrochemical systems. We note that in electrosorption-based processes, there is another definition of selectivity—the separation factor. The separation factor, which is defined as the ratio of the thermodynamic equilibrium partitions of ion in solid phase (electrodes) to the liquid phase (solution) [66], is used as a metric to quantify the selectivity of the electrodes for electrosorption of the target ions.

The widely used commercial CEMs in electrochemical reactors for ammonia recovery include Nafion N117 (Dupont/Ionpower, Inc., New Castle, DE, USA), CMI-7000 (Membrane International, Membranes International, Inc., Ringwood, NJ, USA), CMH-PP Ralex (Ralex Mega, Straz pod Ralskem, Czech Republic), CEM-Type I and CEM-Type II (FUJIFILM Europe, GmbH, Germany), etc. These membranes have been applied in various kinds of processes for ammonia recovery from wastewater. Table 1 lists the properties of several commonly-used commercial CEMs, including the backbone/branches, thickness, electrical resistance, permselectivity, total exchange capacity, water permeability, thermal resistance, and pH resistance. All the CEMs listed in Table 1 use sulfonic acid as fixed functional groups and thus allow cations as counter-ions to transport across the membrane, exhibiting excellent properties in transporting cations and blocking water and anions. The backbone and branches vary from tetrafluoroethylene/perfluorovinyl ether to polyamide. The structure of Nafion N117 CEM was reported as a sandwich. In the middle of the sandwich structure lies the embedded core portion, which is either empty or flooded by water/methanol molecules. In contrast, the outer layers of the sandwich model are consist of polymer chains functionalized by sulfonic acid groups [67,68]. All CEMs listed in Table 1 can be used in a wide range of pH values, making CEM suitable for the electrochemical cell due to the acidic environment in anode compartments and basic electrolytes in the cathode. Previous studies have related the properties of CEM to ammonia recovery performance in electrochemical systems. For example, Dykstra et al. reveal that the charge density of the CEM does not impact the transport number of ammonium ions across the membrane [55]. However, since previous studies used various kinds of membranes and system configurations, there is no uniform conclusion of the impact of membrane properties on the electrochemical processes.

The total exchange capacity represents the amount of total sulfonic acid groups available for ion exchange per weight of dry membrane, expressed in milliequivalents per gram (meq/g). It reflects the amount of cation charges that can be carried and conveyed by the CEM. The total exchange capacity affects the fixed charge density, which describes the amount of fixed sulfonic groups per gram of water in the membrane. In addition, permselectivity, which is defined as the ratio of the transfer of electric charges by specific counterions (i.e., NH_4_^+^) and the total charge transport across the CEM, is another critical parameter of membrane influenced by both the fixed charge density and the total exchange capacity [75,76,77,78,79].

Water permeability describes the transport of water molecules across the membrane. Kingsbury et al. has demonstrated that water transport impacts the permselectivity of both AEMs and CEMs [62]. In electrochemical systems, water transport across CEMs is negligible, probably owing to the minimal osmotic pressure across CEM and the thickness of CEMs. Since CEM is used to separate the electrolytes and transport specific ions, the CEM with low water permeability is favored in electrochemical systems. However, the sulfonic acid groups attached to the polymer chains in the CEMs increase the hydrophilicity of the membranes, which facilitates the water uptake of a CEM. The water uptake of a typical CEM, Nafion N117, is 11.7%, owing to the hydrophilic portions of the membrane as well as the existence of hydrated ionic groups inside the membrane [69].

The performance of the electrochemical systems for ammonia recovery is summarized and listed in Table 2. The max NH_4_^+^-N C_0_ represents the maximum ammonium nitrogen concentration of the influent wastewater. The chemical oxygen demand (COD) of the influent is also listed. The max transport number denotes the highest transport number of ammonium or TAN reached during the electrochemical processes. The efficiency is the ratio of the recovered/removed ammonium to the NH_4_^+^-N C_0_. When landfill leachate is fed into a MEC reactor, 65.7% of ammonium ions can be recovered as ammonium sulfate [80]. With a continuous stirred tank reactor (CSTR) integrated with a submersible microbial desalination cell (SMDC), 40.8% of ammonia in the anaerobic digestate can be recovered [81]. In MES, gas permeable membranes can be integrated to strip out the ammonia from the cathode side and achieve 93% ammonia recovery from urine. In addition, the hydrogen evolution reaction occurring in the cathode can be used as substrate in the anode, forming a hydrogen recycling electrochemical system to reduce energy consumption for ammonia recovery [31].

A pilot-scale ED with 30 pairs of CEM-AEMs could remove 96–100% of ammonia from wastewater and recover a concentrated effluent with an NH_4_^+^-N concentration of 7.1 g/L [43]. Besides, the ED process can be combined with another module to enhance its performance. Liquid-liquid membrane contactors can be integrated with a five cell-pair ED reactor, recovering ammonia directly as liquid fertilizers [45].

In flow-electrode capacitive deionization (FCDI) integrated with a CEM-DF-120 (Tianwei Membrane Technology Co., Ltd., Shandong, China), the efficiency of ammonia removal from municipal wastewater can reach 87% after system optimization [82]. When a gas permeable membrane is integrated into an FCDI reactor, 78% of influent ammonia can be recovered from diluted wastewater [34]. In BES/ECS systems, load ratio, L_N_, which is the ratio of the applied current and the TAN loading rate, has been proposed to assess the optimal conditions of the reactor for ammonia removal efficiency and energy input. When L_N_ is larger than 1, excess current is applied compared to the TAN loading, while L_N_ lower than 1 represents insufficient current for TAN transport. It was found that L_N_ needs to be higher than 1 to achieve the optimal system performance for both synthetic wastewater and urine due to the back diffusion of ammonia from the cathode to the anode compartment [62]. However, load ratio has not been used in electrosorption and ED processes. In the electrochemical process, current and voltage are affected by various parameters, including the rate of the redox reaction at electrodes in redox-driven processes, electrosorption rate in MES, internal resistance, and system design and configurations. Therefore, there is no uniform metrics for all the electrochemical processes as the optimal voltage or current density for ammonia recovery.

## 5. Conclusions and Perspectives

Cation exchange membrane has been extensively applied in electrochemical systems for ammonia recovery from wastewater. In this study, we introduced four kinds of electrochemical systems, including bioelectrochemical systems, electrochemical stripping, electrodialysis, and membrane electrosorption, and discussed the role CEM plays in these processes for ammonia recovery. Besides, the theory used to simulate the ion transport phenomena is also elucidated. The key performance metrics related to ammonia recovery and properties of CEM membrane are then discussed. Despite the progress, there are some challenges we need to address. First, when the electrochemical systems are applied for ammonia recovery, CEMs are in direct contact with ammonia-rich wastewater. Therefore, the degree of ion-ion selectivity is crucial to achieving selective ammonium transport across the membrane. In addition, because of the direct contact of the CEMs and the wastewater, fouling and scaling of the membrane in complex wastewater matrices will be an inevitable and critical issue we need to address in future research. Third, the extraction of dissolved ammonia from the cathode chamber is considered the limiting step for ammonia recovery in electrochemical systems. Hence, we highlight the critical need for incorporation with the ammonia extraction process. Fourth, the energy performance, including energy generation and consumption, of these electrochemical systems for ammonia recovery needs to be systematically analyzed to evaluate if the electrochemical systems using CEM are feasible to recover ammonia from wastewater.

## Figures and Tables

**Table 1:** Properties of commercial cation exchange membranes.

Membrane Name	Company Name	Backbone /Branches	Thickness	Electrical Resistance	Permselectivity	Total Exchange Capacity	Water Permeability	Thermal Resistance	pH Resistance	Reference
			mm	Ohm·cm^2^		meq/g	mL/h/m^2^	°C		
Nafion N117	Dupo-nt	tetrafluoroethylene/perfluorovinyl ether	0.183	1.725	100%	0.9	n.a. *	n.a.	n.a.	[68,69,70]
CMI-7000	Membrane Intl	polystyrene/divinylbenzene	0.45	30	94%	1.6	32 at 0.34 bar	90	1–10	[71]
CMH-PP- Ralex	Mega	polyester/polyethylene	0.45	8	90%	n.a.	0 at 1 bar	65	0–14	[72]
CEM-Type I	FUJIFILM	Polyamide	0.135	2.7	92%	n.a.	13 per bar	40	4–12	[73,74]
CEM-Type II	FUJIFILM	Polyamide	0.16	8	96%	n.a.	3.5 per bar	40	4–12	[73,74]

*: Not applicable.

**Table 2:** Summary of performances of electrochemical systems.

Membrane Name	System Type	Max Current Density or Voltage	Wastewater Type	Max NH_4_^+^-N C_0_, gN/L	COD, g/L	Max Transport Number	Recovery/Removal	Efficiency	Reference
Nafion N117	BES	0.5 A/m^2^	Synthetic urine	4.05	0.6	n.a. *	Recovery	11.4%	[25]
Nafion N117	BES	0.176 A/m^2^	Synthetic wastewater	37.9	0.0966	n.a.	Removal	63.7%	[83]
CMI-7000	BES	50 A/m^2^	Synthetic urine	5.88	7.36	n.a.	Recovery	49.5%	[84]
CMI-7000	BES	0.6 A/m^2^	Activated sludge inculated wastewater	n.a.	n.a.	0.9	Removal	85%	[85]
CMI-7000	BES	7.6 A/m^2^	Synthetic ammonia rich wastewater	6	n.a.	n.a.	Recovery	88%	[81]
CMI-7000	BES	0.72 A/m^2^	Landfill leachate	4.54	9.175	n.a.	Recovery	66%	[80]
CMI-7000	BES	0.8 V	Synthetic wastewater	5.111	2	n.a.	Recovery	99.7%	[86]
CMH-PP Ralex	BES	0.917 A/m^2^	Synthetic wastewater	4	0.64	n.a.	Recovery	7%	[20]
Nafion N117	BES	93.8 A/m^2^	Real livestock wastewater	3	30	0.492	Removal	73%	[7]
CMI-7000	BES	4.33 A/m^2^	Synthetic ammonia rich wastewater	6	2	n.a.	Recovery	40.8%	[87]
CMI-7000	BES	1.8 A/m^2^	Synthetic wastewater	1.19	3.23	0.489	Removal	82%	[29]
CMH-PP Ralex	BES	1.7 A/m^2^	Diluted urine	0.5	n.a.	0.7	Recovery	49%	[88]
Nafion N117	ECS	50 A/m^2^	Synthetic wastewater/urine	3.92	n.a.	1	Removal	94%	[64]
Ultrex CMI-700	ECS	50 A/m^2^	Synthetic urine	8.02	n.a.	0.55	Recovery	57%	[89]
CMI-7000	ECS	48 A/m^2^	Urine/synthetic urine	5.49 ± 0.53	n.a.	n.a.	Removal	91.6 ± 2.1%	[90]
CMI-7000	ECS	100 A/m^2^	Urine/synthetic urine	4	n.a.	n.a.	Recovery	93%	[8]
CMI-7000	ECS	100 A/m^2^	Synthetic wastewater	0.03, 0.3, 3	n.a.	n.a.	Recovery	65%	[26]
Nafion N117	ECS	50 A/m^2^	Urine/synthetic urine	4	n.a.	0.56	Recovery	73%	[31]
CEM-Type II	ED	7 V	Activated sludge inculated wastewater	4	n.a.	n.a.	Recovery	89.6%	[45]
JCM-II-07	ED	62 V	Synthetic wastewater	0.6	n.a.	n.a.	Removal	95.8–100%	[91]
CR67	ED	38 V	Centrate from WWTP **	0.847 ± 0.391	0.316 ± 0.044	0.4	Removal	96–100%	[43]
IONAC^®^ MC-3470	ED	13.4 A/m^2^	Final effluent of WWTP	37.04 ± 0.02	n.a.	n.a.	Removal	95–98%	[92]
SK MVK and SC	ED	6–10 V, 45.3 A/m^2^	Synthetic wastewater	0.5	n.a.	n.a.	Recovery	63.2%	[93]
SK	ED	n.a.	Synthetic wastewater	1.5	n.a.	0.69	Removal	85–91%	[94]
CEM-DF-120	MES	1.2 V	Synthetic wastewater	0.7	n.a.	n.a.	Recovery	63%	[34]
CEM-DF-120	MES	1.2 V	Synthetic wastewater	0.02	n.a.	0.87	Removal	87.00 ± 0.79%	[82]
CEM-Type I	MES	17.2 A/m^2^	Synthetic wastewater	0.04	n.a.	n.a.	Recovery	78%	[95]
CEM-Type I	MES	10.4 A/m^2^	Synthetic wastewater/urine	0.043	n.a.	0.75	Recovery	77.8%	[96]
CEM-Type II	MES	6.8 A/m^2^	Synthetic wastewater	0.043	n.a.	n.a.	Recovery	74.7%	[97]
CEM-Type II	MES	6 A/m^2^	Synthetic wastewater	0.043	n.a.	n.a.	Recovery	32%	[98]
Neosepta CMX	BPMED-MES	300 A/m^2^	Synthetic wastewater	0.175	n.a.	n.a.	Removal	77%	[41]
CR67	BPMED	30 V	Centrate from WWTP	1.189 ± 0.032	n.a.	0.86	Recovery	88.4%	[49]
CMB	BPMED	500 A/m^2^	Synthetic wastewater	28	n.a.	0.9	Removal	96%	[99]
PE 001	BPMED	480 A/m^2^	Synthetic wastewater	28.8	n.a.	0.8	Recovery	43.75%	[52]

*: Not applicable; **: Wastewater treatment plant.

## Data Availability

Data sharing not applicable.
